# Bacteria Invade the Brain Following Sterile Intracortical Microelectrode Implantation

**DOI:** 10.21203/rs.3.rs-3980065/v1

**Published:** 2024-03-07

**Authors:** Jeffrey Capadona, George Hoeferlin, Sarah Grabinski, Lindsey Druschel, Jonathan Duncan, Grace Burkhart, Gwendolyn Weagraff, Alice Lee, Christopher Hong, Meera Bambroo, Hannah Olivares, Tejas Bajwa, William Memberg, Jennifer Sweet, Hoda Amani Hamedani, Abhinav Acharya, Ana Hernandez-Reynoso, Curtis Donskey, George Jaskiw, Ricky Chan, A. Ajiboye, Horst von Recum, Liangliang Zhang

**Affiliations:** Case Western Reserve University; Case Western Reserve University; Case Western Reserve University; Case Western Reserve University; Case Western Reserve University; Case Western Reserve University; University of Florida; Case Western Reserve University; Case Western Reserve University; Case Western Reserve University; Case Western Reserve University; Case Western Reserve University; Case Western Reserve University; Case Western Reserve University; Case Western Reserve University; Case Western Reserve University; University of Texas at Dallas; Case Western Reserve University; Case Western Reserve University; Institute for Computational Biology, Case Western Reserve University; Case Western Reserve University; Case Western Reserve University; Case Western Reserve University

## Abstract

Brain-machine interface performance is largely affected by the neuroinflammatory responses resulting in large part from blood-brain barrier (BBB) damage following intracortical microelectrode implantation. Recent findings strongly suggest that certain gut bacterial constituents penetrate the BBB and are resident in various brain regions of rodents and humans, both in health and disease. Therefore, we hypothesized that damage to the BBB caused by microelectrode implantation could amplify dysregulation of the microbiome-gut-brain axis. Here, we report that bacteria, including those commonly found in the gut, enter the brain following intracortical microelectrode implantation in mice implanted with single-shank silicon microelectrodes. Systemic antibiotic treatment of mice implanted with microelectrodes to suppress bacteria resulted in differential expression of bacteria in the brain tissue and a reduced acute inflammatory response compared to untreated controls, correlating with temporary improvements in microelectrode recording performance. Long-term antibiotic treatment resulted in worsening microelectrode recording performance and dysregulation of neurodegenerative pathways. Fecal microbiome composition was similar between implanted mice and an implanted human, suggesting translational findings. However, a significant portion of invading bacteria was not resident in the brain or gut. Together, the current study established a paradigm-shifting mechanism that may contribute to chronic intracortical microelectrode recording performance and affect overall brain health following intracortical microelectrode implantation.

## Introduction

Intracortical microelectrodes hold promise for studying brain functions and treating neurological disorders by recording neural signals from the brain^1,2^. However, translation of this technology to clinical applications requires long-term reliability of the microelectrodes^3^. The neuroinflammatory response in the brain following implantation has been identified as a major factor influencing microelectrode performance^3–5^. Despite extensive studies on the identification of triggers of neuroinflammation and their related pathways following microelectrode implantation^4,6–10^, limited information exists on neuroinflammatory responses to microelectrodes associated with the presence of bacteria at the site of the implant^11^.

Bacteria can enter the brain at various stages of device implantation during the surgical procedure, ranging from contamination of the initially sterile device to transport by blood to the implantation site^12,13^. We have previously demonstrated that bacterial contamination from the implant itself can be avoided with rigorous sterilization and proper surgical technique^11^, and decades of similarly rigorous sterilization and proper surgical techniques in human participants have reported no adverse related bacterial contamination events^14^. Degradation of blood-brain barrier (BBB) integrity is an appreciable consequence of microelectrode-mediated neuroinflammation and increases the entry of blood-borne components into the brain, where they could amplify and extend the neuroinflammatory response^3,4,6,15–18^. The integrity of the mucosal lining of the intestines is dysfunctional following brain injury^19^, indicating a potential pathway for gut-derived bacteria to enter the brain following the trauma associated with microelectrode implantation. The investigation of microbes in diseased and injured brains has recently become an exciting area of research^20,21^. Publicly presented data, which have not appeared in a peer-reviewed publication, suggested that certain gut bacterial constituents could penetrate the BBB, become resident in brain parenchyma in rodents and humans, and play a role in health and disease^21^. In fact, there is robust evidence that gut microbiota can trigger and mediate systemic neuroinflammatory processes that have been implicated in neuropsychiatric conditions such as schizophrenia, depression, anxiety, Alzheimer’s, Parkinson’s, and stroke^20,22,23^. Despite such links, there are no reports of how brain responses to gut microbiome infiltration following microelectrode implantation might be explored to improve device tissue integration and performance.

The gut microbiome affects innate and adaptive immunological players, ranging from epithelial cells and antigen-presenting cells to innate lymphoid cells and regulatory T-cells^24,25^. Diverse microbiota-derived bioactive molecules, including bacteria-produced metabolites and even neurotransmitters, have been strongly implicated in inflammatory processes in the gut and the brain^26^. However, the role of gut-resident microorganisms translocated beyond the gut’s usual niche remains unclear. There are multiple elements and processes through which the gut microbiome affects brain health that constitute the microbiome-gut-brain axis in both acute and chronic brain disease^27^.

Our study explores the role of gut-derived bacteria infiltration of the brain tissue in contributing to the neuroinflammatory response following intracortical microelectrode implantation in a mouse model. The current study was designed to test the hypothesis that microelectrode implantation could amplify dysregulation of the microbiome-gut-brain axis. Utilizing 16S rRNA gene sequencing, we have confirmed the presence of gut-resident bacteria and bacteria of an undefined origin in the mouse brain, which changes composition following microelectrode implantation. We also demonstrated that systemic antibiotic treatment altered the concentration and composition of microbes in both feces and brain tissue. Manipulations of the microbiome with antibiotic treatment were associated with changes in single-unit recordings using intracortical microelectrodes, which corresponded to temporal changes in the neuroinflammatory response as indicated through spatial proteomics and spatial transcriptomics. Our results suggest that modulating the invasion of microbes into the brain may impact microelectrode performance to improve quality and stability.

## RESULTS

### Bacteria Invade the Brain After Intracortical Microelectrode Implantation

Microbiome composition can be measured directly by sequencing the 16S rRNA gene from bacterial DNA isolated from feces or other tissue^28^. Therefore, our first step was to identify and compare the composition of microbes present in both fecal matter and the brain tissue of naïve unimplanted mice and microelectrode-implanted mice.

**Figure 1:**
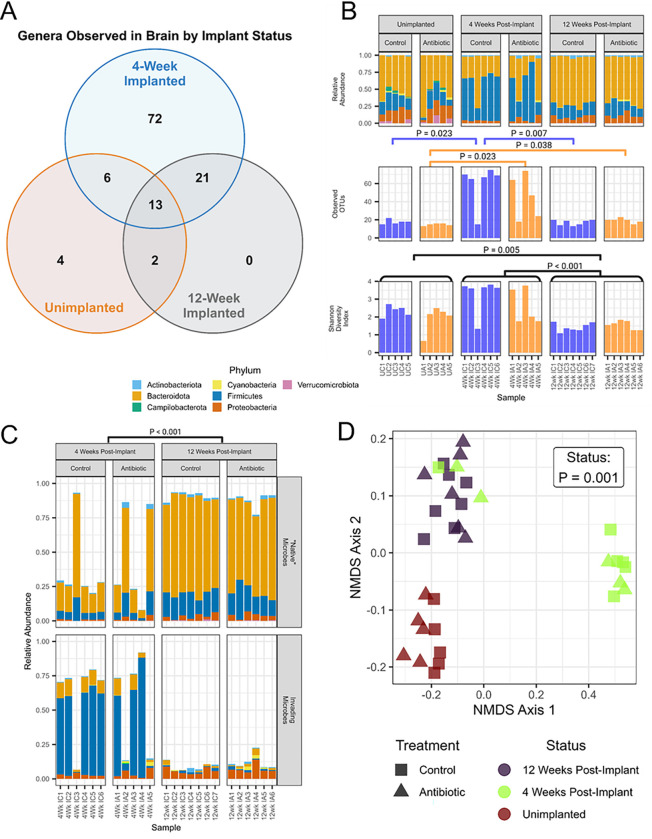
Microelectrode Implantation and Antibiotic Treatment Influence Invasive Microbe Diversity and Composition in the Brain. Short-term and long-term differences in microbial presence and abundance were observed in the brain following implantation; while systemic antibiotic treatment caused moderate acute disruption. (A) Venn diagram showing the number of genera detected in the brain samples of control animals that were unimplanted or at 4- or 12-weeks post-implantation. (B) Bar plots detailing the relative abundance of bacteria across all groups by phylum, number of observed OTUs, and Shannon Diversity Index. (C) The relative abundance of native microbes (detected in the unimplanted brain) and invading microbes (not detected in the unimplanted brain) for the control and antibiotic-treated animals at 4- and 12-weeks post-implantation. (D) A non-metric multidimensional scaling (NMDS) approach for comparing the composition of bacteria for each time point and treatment group.

Here, the V3-V4 region of the gene for the 16S rRNA small subunit was sequenced using bacterial DNA extracted from biopsy punches of unimplanted non-treated control brains 2 weeks after housing separation. Similar sequences were assigned to operational taxonomic units (OTUs, representing species-level observations) and their read counts were used to compare microbial composition between unimplanted brains and implanted control brains both 4- and 12-weeks post-implantation (Fig. 1). Variation in microbial communities manifests primarily via differences in prevalence (presence/absence of an OTU in a sample) and/or differences in abundance (proportion of sample reads derived from an OTU). Significant differences in the prevalence and the abundance of microbes were observed in the brain following microelectrode implantation. Observations at the genus level will also be discussed as changes to the genera.

Across all samples within a group, brain tissue from the unimplanted control group contained 25 total genera (four unique), whereas the 4-week control group contained 112 total genera (72 unique, 93 invading), and the 12-week control group contained 36 total genera (zero unique, 21 invading) (Fig. 1A). Nineteen of the 25 genera found in the unimplanted brain were found in the mouse brain tissue 4 weeks after microelectrode implantation, while an additional 93 gut-derived genera were found in the brains of implanted mice 4 weeks after microelectrode implantation. By 12-weeks post-implantation, 72 of the 93 invading genera were no longer found in implanted brain tissue, while 21 of the invading genera could still be detected in the brain. Interestingly, 13 genera found in the unimplanted brains were also found at both time points post-implantation, while only two of the original genera found in the unimplanted brains were able to repopulate the brain after becoming absent for some duration post-implantation (Fig. 1A).

### Antibiotic Treatment Facilitates Invasive Microbe Diversity

An additional cohort of mice was treated with antibiotics to deplete fecal (gut) microbiota. Mice with differential expression of microbes could then be used to examine the correlation between the composition of the microbes invading the brain and microelectrode recording performance. Antibiotic-treated mice were provided with an antibiotic cocktail of Ampicillin, Clindamycin, and Streptomycin in their drinking water following established protocols^29^. Antibiotic-treated mice displayed significant alterations to the gut microbiome as early as one week after the start of treatment, which continued throughout the study (**Supplemental Fig. S1A-C**). Antibiotic treatment had no discernible effect on microbial composition in the unimplanted brain (**Supplemental Fig. S1D**).

Firmicutes was the most abundant phylum found in the brain at 4-weeks post-implantation, with Bacteroidota dominating the unimplanted and 12-weeks post-implantation brains (Fig. 1B). Linear discriminant analysis Effect Size (LEfSe) is a method used in biomarker discovery to identify taxa most likely to be associated with differences between experimental groups^30^. Higher values of the log-linear discriminant analysis score indicate greater enrichment of taxa within a particular group (**Supplemental Fig. S2**). The unimplanted brain was characterized by an abundance of microbes from the phyla Bacteroidota (genus Muribaculaceae) and Firmicutes (genus Lactobacillus). The 4-week post-implantation brain was characterized by an abundance of microbes from the phylum Firmicutes, from the class Clostridia. The 12-week post-implantation brain was characterized by an abundance of microbes from the phyla Bacteroidota (genus Bacteroides) and Proteobacteria.

The Shannon Diversity Index, a measure of alpha (within a sample) diversity, provides a quantitative assessment of the species richness and evenness of a bacterial community sample; it is robust to sample composition, with a higher value indicating greater sample diversity^31^. The Shannon Diversity Index varied significantly by implantation status but not by treatment group, with the highest values observed in the 4-week post-implantation group (Fig. 1B). The number of observed OTUs varied significantly within treatment group with the largest number of OTUs observed at 4 weeks, in which 60 ± 22 OTUs were observed in rarefied control brains and 45 ± 24 OTUs in rarefied antibiotic brains (Fig. 1B). At 12 weeks post-implantation, control brains contained 17 ± 3 OTUs and antibiotic contained 19 ± 3 OTUs, compared to 18 ± 3 OTUs in unimplanted control brains and 15 ± 1 OTUs in unimplanted antibiotic brains (Fig. 1B).

OTUs were then classified as “native” (detected in the unimplanted brain) or invading (not detected in the unimplanted brain) to investigate differences in abundance and taxonomy. At 4-weeks post-implantation, invading microbes composed 62.8% ± 27.7% of the relative abundance in the control group (*n* = 6) and 54.1% ± 37.1% of the relative abundance in the antibiotic group (*n* = 5) (Fig. 1C). At 12-weeks post-implantation, invading microbes made up 8.9% ± 2.8% of the relative abundance in controls (*n* = 7) and 12.1% ± 5.4% of the relative abundance in the antibiotics group (*n* = 6) (Fig. 1C). The populations varied significantly based on implantation status. The most abundant invading microbes were from the phylum Bacteroidota in the unimplanted group, Firmicutes at 4 weeks, and Proteobacteria at 12 weeks. The invading Firmicutes bacteria were largely gone by 12 weeks.

Given the complexity of comparing multiple groups with feature-rich microbial data, we applied the dimension reduction technique of non-metric multidimensional scaling (NMDS) to visualize the relationship between samples across the three implantation statuses (unimplanted, 4-weeks, and 12-weeks post-implantation) and two treatment groups (control vs. antibiotic-treated) in 2-dimensional space (Fig. 1D). The unweighted UniFrac distance measures Beta (between-groups) diversity by considering the phylogenetic information of observed microbes^32^. We used the UniFrac distance to quantify the degree of difference between samples by calculating the fraction of branch length in the de novo-assembled phylogenetic tree that is unique to either of the two samples being compared. Samples that are more like each other have fewer unique evolutionary relationships and appear closer together in the 2-dimensional ordination space, as will experimental groups. Here, we found three significantly distinct clusters, largely segregated by implantation status, suggesting that the brains of unimplanted, 4-week post-implanted, and 12-week post-implanted animals have distinct microbial populations (Fig. 1D). Two antibiotic-treated and one control brain from the 4-week post-implantation are like that of the 12-week group, suggesting that there is variation in the rate at which the brain-bacterial environment may stabilize. Of note, none of the implanted brains we examined returned to a bacterial composition resembling the unimplanted brain tissue, regardless of treatment or time point.

### Correlation Between Gut and Brain Isolated Microbes

**Figure 2:**
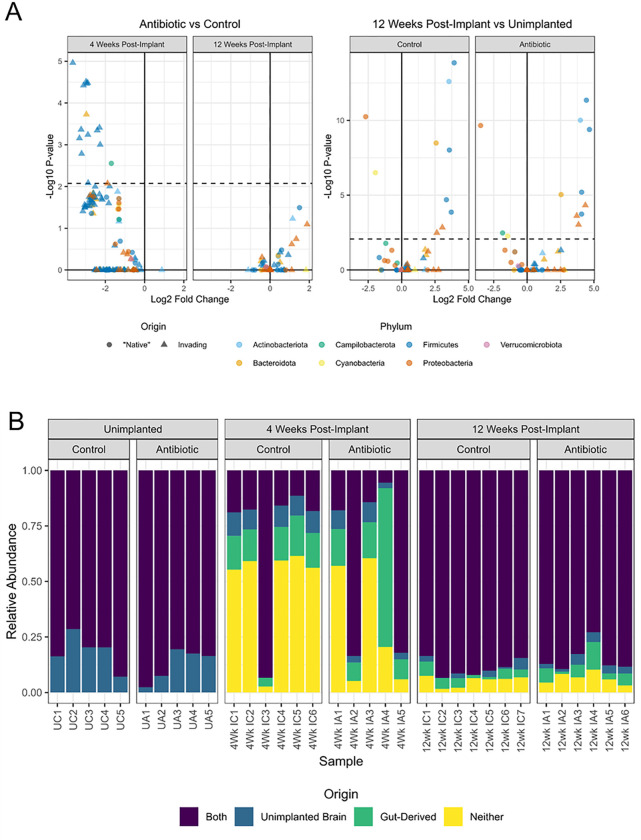
Antibiotic treatment influences specific types of bacteria that enter the brain. (A) Analysis of Composition of Microbiomes with Bias Correction (ANCOM-BC) to identify differential abundance of microbes by implantation status and treatment group. (B) The relative abundance of bacteria composition across all groups to understand the origin of the present bacteria. Bacteria origins are classified as gut-derived (only observed in the gut), unimplanted brain (only observed in the unimplanted brain), both (observed in both the gut and the unimplanted brain), or neither (observed in neither the gut nor the unimplanted brain).

Given that daily administration of antibiotics via drinking water altered the composition of microbes in the fecal matter (**Supplemental Fig. S1A-C**), we next examined the composition of microbes in the brain tissue for microelectrode implanted mice. Analysis of Compositions of Microbiomes with Bias Correction (ANCOM-BC) was used to model microbial communities via a linear regression framework and test for differential abundance by implantation status (unimplanted, 4-weeks and 12-weeks post-implantation) and treatment group (antibiotic, control). Notably, there were 16 genera found at lower abundance in the antibiotic-treated brain at 4-weeks post-implantation, 15 of which were not detected in the unimplanted brain (Fig. 2A). In addition, 13/16 genera were from the class Clostridia of the phylum Firmicutes, found in the tissue adjacent to microelectrodes in this study. At 12-weeks post-implantation, we found there to be no differentially abundant genera between antibiotic-treated and control brains (Fig. 2A).

Fecal and brain bacteria were compared to understand the origin of invading bacteria. There were 198 OTUs observed in the implanted brain, of which only 32 were detected in the unimplanted brain. Of the 166 OTUs invading the brain, 49 were observed in the gut samples, lending credibility to the hypothesis that gut-derived bacteria invade the brain after IME implantation. However, these 49 gut-derived bacteria represent only a portion of the invading microbes, suggesting that the remaining majority come from a different source in the body’s microbiome outside of the gut and brain (Fig. 2B). At 4 weeks post-implantation in the control group, 13.7% ± 4.9% of total reads (27.7% ± 16.0% of invading reads) were gut-derived and 49.0% ± 22.8% of total reads (72.3% ± 16.0% of invading reads) were of unknown origin. In the 4-week antibiotic-treated group, 24.3% ± 26.7% of reads (48.5% ± 25.4% of invading reads) were gut-derived and 29.8% ± 27.1% (51.5% ± 25.4% of invading reads) were of unknown origin. At 12 weeks post-implantation in the control group, 3.7% ± 1.9% of reads (42.1% ± 22.6% of invading reads) were gut-derived and 9.0% ± 2.8% (57.9% ± 22.6% of invading reads) were of unknown origin, while for the antibiotic group, 5.6% ± 3.9% of reads (44.1% ± 19.7% of invading reads) were gut-derived and 12.1% ± 5.4% of reads (55.8% ± 19.7% of invading reads) were of unknown origin.

### Antibiotic Treatment Impacts Intracortical Microelectrode Performance

Functional, single-shank, silicon 16-channel intracortical microelectrodes were implanted into the primary motor cortex to obtain awake neural recordings. Animals were separated into two cohorts consisting of untreated control and antibiotic-treated groups. Biweekly recordings and analysis indicate that arrays implanted in antibiotic-treated animals performed significantly better than the control group based on measurement of the proportion of active electrodes, or active electrode yield (AEY), at week 0 (day of implantation), week 1, week 4, and week 5 (Fig. 2A). The largest difference in AEY was observed in week 4 (79% for antibiotic-treated animals vs. 62% for the control group).

Antibiotic-treated and control animals declined significantly in performance over time, consistent with historical data (Fig. 2B)^4,34,35^. When grouped into known phases for the maturation of the neuroinflammatory response^36,37^, antibiotic-treated mice performed significantly better in the acute (weeks 0–5) phase of implantation (80% AEY for antibiotics vs. 67% AEY for control), exhibited no difference during the sub-chronic (weeks 6–11) phase (52% for antibiotics vs 54% for control), and displayed a significant decline in performance at the chronic (week 12) time period (42% for antibiotic vs 56% for control, Fig. 2B).

During the sub-chronic implant period, the peak-to-peak voltage (V_pp_) (97.9 μV ± 43.7 μV for antibiotic vs. 81.1 μV ± 34.1 μV for control, Fig. 2C), noise levels (12.4 ± 2.7 μV μV for antibiotic vs. 11.3 μV ± 2.7 μV for control, Fig. 2D), and spike rate (12.0 ± 17.8 for antibiotic vs. 6.8 ± 6.9 for control, Fig. 2E), were all significantly higher in the antibiotic group compared to the control. SNR showed no significant change across groups or time points (Fig. 2F).

### Antibiotic Treatment Impacts the Neuroinflammatory Response to Intracortical Microelectrodes

Neuroinflammation has long been associated with intracortical microelectrode failure^3,16,38^. Here, we utilized one of the most advanced methods reported to date to assess the intracortical microelectrode-tissue interface, both spatial proteomics (with and without cell specificity) ^39^, and spatially-resolved whole mouse transcriptomics^40,41^. Our goal was to begin to understand the potential relationship between invasive microbes in the brain, neuroinflammation, and microelectrode recording performance.

Spatial and cell-specific neural proteomic evaluation of the implant site (up to 270 μm from the implant) provides a robust view of the health of the brain tissue and the effect of treatment on inflammation. Comparisons between antibiotic and control were made at 4- and 12-weeks post-implantation, along with temporal comparisons within the antibiotic and control groups (4-week antibiotic: *n* = 4, 4-week control: *n* = 3, 12-week antibiotic: *n* = 3, 12-week control: n = 3). All comparisons between antibiotic and control were made with control as the baseline. A negative fold change indicates lower expression in antibiotic-treated mice compared to the control group (“downregulation”), while a positive indicates higher expression in antibiotic-treated mice compared to the control group (“upregulation”). Across all comparisons, 28 of the 39 possible proteins were differentially expressed in at least one comparison. Table 1 shows the full list of 39 proteins examined and the 6 proteins used for quality control and normalization. The complete area of interest (AOI) represents the tissue within 270 μm of the implant site. Within the AOI, the inner AOI is the tissue adjacent to the implant site to 90 μm from the implant site; the middle AOI is the tissue 90 μm to 180 μm from the implant site; and the outer AOI is the tissue 180 μm to 270 μm from the implant site. Neuron (NeuN-positive) and astrocyte (GFAP-positive) cell-specific regions were analyzed for each AOI. All twelve potential combinations of the AOIs used for comparison in this study can be visualized in Fig. 7A and are summarized in Table 2 (See Methods for an in-detail explanation).

**Figure 3:**
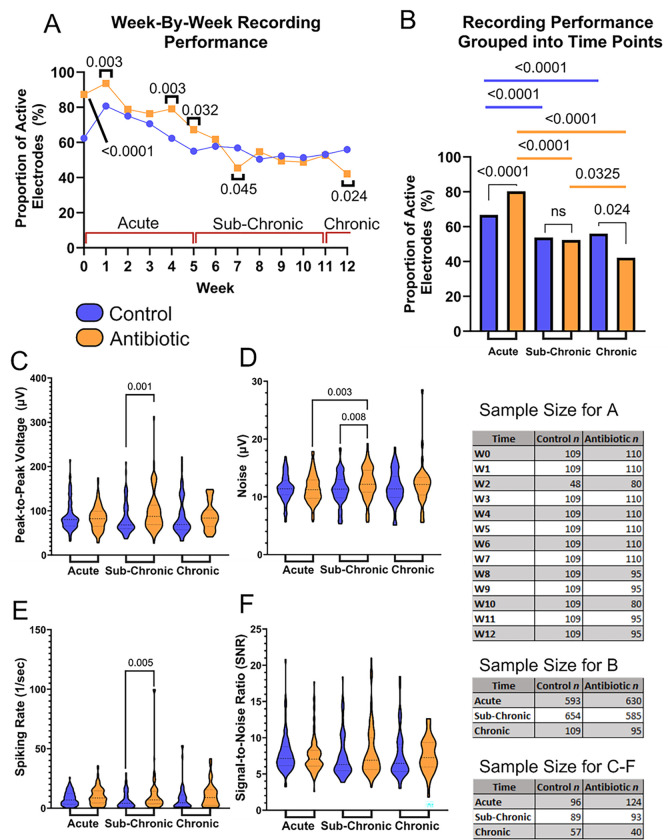
Antibiotic Treatment Significantly Improves the Recording Performance of Intracortical Microelectrodes. Neurophysiological recordings to evaluate the performance of our intracortical microelectrodes and the impact of antibiotic treatment compared to control. Comparisons are made to evaluate (A) the week-by-week proportion of active electrodes and (B) the acute, sub-chronic, and chronic grouped proportion of active electrodes. Additional metrics were evaluated to measure the (C) peak-to-peak voltage (V_pp_), (D) root-mean-squared of the noise, (E) spike-rate of the single units, and (F) signal-to-noise ratio (SNR) of all active channels. The sample size for all comparisons is included as well. Statistically significant p-values are displayed in the figure. No symbol indicates a lack of statistical significance. No comparisons were made between antibiotic and control at differing time points.

The proteomic analysis of antibiotic-treated mice compared to control at 4-weeks post-implantation indicated that in all cases of differential protein expression, the proteins were decreased in expression in tissue from the antibiotic-treated mice compared to the untreated control group (Fig. 3, Table 2). Seven of the twelve AOI comparisons indicated differential protein expression (Fig. 3). Without cell-specific segmentation of the AOI, the complete AOI (0–270 μm), inner, and outer AOI all showed differential protein expression. Specifically, protein expression of our panel showed the downregulation of 18 proteins for the full AOI (ATG12, ATG5, BAG3, CD163, CD31, CD40, CD68, CSF1R, MAP2, NeuN, NfL, OLIG2, P62, PLA2G6, SYP, TMEM119, ULK1, and VIM), six in the inner region (ATG12, CD68, MAP2, SYP, TMEM119, ULK1), none in the middle, and 19 proteins in the outer region (ATG12, CD31, CD40, CD45, CD68, CSF1R, CTSD, GPNMB, ITGAX, MAP2, NeuN, NfL, P62, PLA2G6, SYP, TMEM119, ULK1, VIM, and VPS35) (Fig. 3A–D, Table 2).

For neuron-specific comparisons, there were 15 total proteins downregulated for the full AOI (ATG12, BAG3, CD31, CD68, CSF1R, GPNMB, MAP2, NfL, OLIG2, P62, PLA2G6, SYP, TMEM119, ULK1, and VIM), none in the inner region, 18 downregulated in the middle region (ATG12, BAG3, CD11b, CD31, CD39, CD45, CD68, GPNMB, Ki-67, MAP2, OLIG2, P62, PLA2G6, SYP, TFEB, ULK1, VIM, and VPS35), and eight downregulated in the outer region (BAG3, CD31, CD68, CSF1R, GPNMB, MAP2, SYP, and ULK1) (Fig. 3E–H, Table 2).

In astrocyte-specific comparisons, no proteins were differentially expressed in the full AOI, middle, or outer regions (Fig. 3I–L). However, seven proteins were downregulated in the inner region of the astrocyte-specific AOI (ATG12, CD40, CD68, MAP2, MerTK, TFEB, and ULK1) (Fig. 3I–L, Table 2). Table 2 summarizes comparisons including all twelve AOIs at 4-weeks post-implantation.

The proteomic analysis of brain tissue from antibiotic-treated mice compared to control at 12-weeks post-implantation indicated only one differentially expressed protein, CD163, which is a marker that indicates the transition from pro-inflammatory M1 to M2 anti-inflammatory macrophage phenotype (**Supplemental Fig. S3**) ^42^. CD163 was indicated to be upregulated in the antibiotic-treated group, compared to the untreated control group in the astrocyte-specific collection, in the area between 180–270 μm from the microelectrode-tissue interface. This upregulation to the M2 phenotype may promote preservation of viable neural tissue near the implant site ^43^.

Within treatment groups, examination of temporal changes in protein expression from 4-weeks to 12-weeks post-implantation showed that five of the twelve AOI comparisons of antibiotic-treated mice indicated differential protein expression (**Supplemental Fig. S4, Supplemental Fig. S1**). Two of the more noteworthy comparisons were identified within examinations of the inner ring. There were eight differentially expressed proteins in the astrocyte-specific inner ring, and ten differentially expressed proteins in the non-specific inner ring, suggesting the largest differential expression between the temporal comparison of the antibiotic-treated animals to be in the tissue closest to the microelectrode implant site. Temporal comparison of the untreated control mice also demonstrated that five of the twelve comparisons indicated differential protein expression (**Supplemental Fig. S5, Supplemental Fig. S2**). However, with the untreated control mice, the two groups with the largest differential expression only indicated three or four differentially expressed proteins each, with the remaining comparisons only showing one differentially expressed protein each. The spatial organization of differential protein expression was evenly distributed between AOIs, one total, two inner, one middle, and one outer AOI each demonstrated differential protein expression.

**Figure 4:**
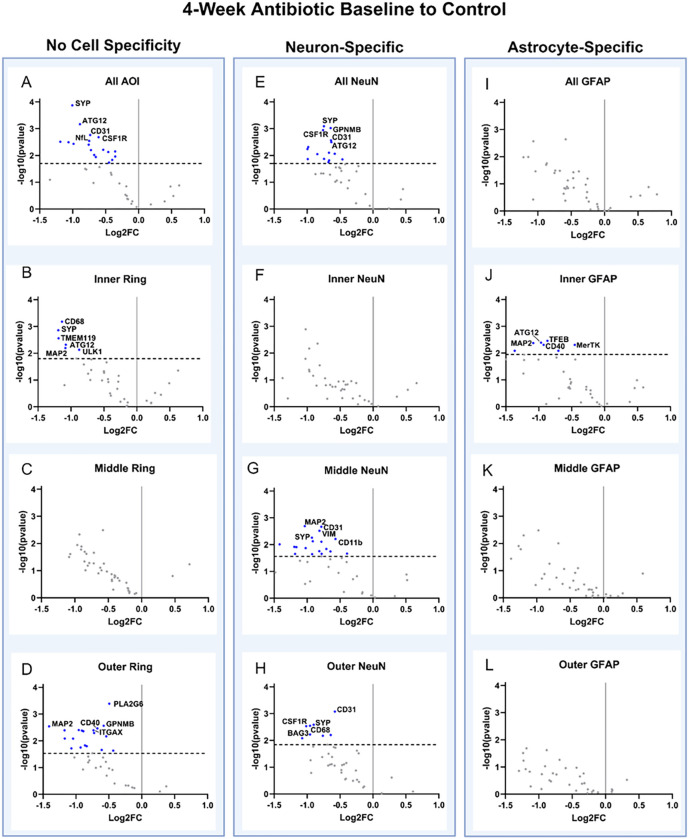
Spatial Proteomic Response is Treatment Dependent at 4-Weeks Post-Implantation. Volcano plots showing neural proteomic panel evaluation of 4-week antibiotic compared to 4-week control across the entire AOI (All, within 270 mm from the implant site), the inner ring of the AOI (Inner Ring, within 0 – 90 mm from the implant site), the middle ring of the AOI (Middle Ring, 90 – 180 mm from the implant site), and the outer ring (Outer Ring, 180 – 270 mm from the implant site) for all cells, all neuron-specific cells (stained using a NeuN antibody), and all astrocyte-specific cells (stained using a GFAP antibody). Proteins with a negative Log2FC indicate downregulation in antibiotic compared to control, while a positive Log2FC indicates upregulation in antibiotic compared to control. Unadjusted p-values are plotted and shown, but all statistical comparisons were done using adjusted p-values. The black dotted line indicates significance (p_adjusted_ = 0.05). Each point on the volcano plot indicates a singular protein, with select proteins shown in the text. (A) through (D) show all cell types across implant regions. (E) through (H) shows only neuron-specific cell comparisons across all implant regions. (I) through (L) shows only astrocyte-specific cell comparisons across all implant regions. A few insignificant proteins were excluded from the plots due to high log_2_(FC) values, causing skewing and making visual representation difficult. Only five significantly differentially expressed proteins were labeled due to space. Refer **Table 2** to for the full list of significantly differentially expressed proteins.

Spatial transcriptomic evaluation of the implant site was performed to understand how many genes were differentially expressed and involved in which pathways and molecular processes. Very few studies have been performed with spatial transcriptomic analysis of the intracortical microelectrode-tissue interface to date^40,41^. However, the NanoString GeoMx system used here is uniquely capable of collecting the entire tissue of interest, rather than orientated spherical regions forming a grid within the tissue being analyzed. Here, the whole mouse transcriptome was first filtered using quality control steps in the NanoString GeoMx software (see Methods for details on filtering) leaving a total of 8259 genes. Of the 8259 genes included in our analysis, 490 were differentially expressed at 4-weeks post-implantation, and 1375 genes were differentially expressed at 12-weeks post-implantation (Fig. 4A–B). Out of all differentially expressed genes, only 52 are shared between the 4- and 12-week time points, indicating consistent temporal changes.

To further our understanding of the response to altering the composition of the brain-invasive gut microbiome and the implications on neuroinflammation and brain health, we completed a pathway analysis using the iPathways software. The differential gene expression detected in our study implicated dozens of biological pathways and functions related to neural health. Here, for brevity and focus, we only discuss pathways in which a high proportion of genes associated with the pathway were differentially expressed at either 4-weeks or 12-weeks post-implantation, or pathways in which many differentially expressed genes switched between up- and down-regulated between the 4- and 12-week timepoints.

At 4-weeks post-implantation, there were 20 differentially upregulated genes of 122 genes associated with ribosomal protein function in antibiotic-treated animals compared to control (Fig. 4C, **Supplemental Fig. 6A**). At 12-weeks post-implantation there were 19 differentially expressed genes in the same pathway. However, at 12-weeks post-implantation only six genes were upregulated and 13 were downregulated in the antibiotic-treated animals compared to the control (Fig. 4D, **Supplemental Fig. 6B**).

Neurodegeneration is an important pathway to consider as it relates to long-term neural health. Healthy, firing neurons can only be detected within ~ 150 μm from the intracortical microelectrode site^44^. Consequently, evidence of neurodegenerative pathways near the implant site detected by spatial transcriptomic analysis is of prominent interest. At 4-weeks post-implantation, there were 17 differentially expressed genes associated with the neurodegenerative pathway (Fig. 4C), with 49 differentially expressed genes at 12-weeks post-implantation. At 4-weeks post-implantation, four genes are differentially expressed in ubiquitin-proteasome system (UPS) disruption (three upregulated, one downregulated, **Supplemental Fig. 7A**), and five genes are upregulated in the mitochondrial dysfunction pathway (**Supplemental Fig. 7B**) (Fig. 4C). At 12-weeks post-implantation, 10 genes associated with UPS disruption were differentially expressed (eight upregulated, one downregulated, Supplemental Fig. S1), 23 genes associated with mitochondrial dysfunction and mitophagy were differentially expressed (15 downregulated, eight upregulated, **Supplemental Fig. 8B**), and six associated with tau protein accumulation were differentially expressed (five upregulated, one downregulated, **Supplemental Fig. 8C**) (Fig. 4D). In addition to pathway analysis, gene ontology (GO) offers added insight into the effect of microbiome alteration via antibiotic treatment on cellular and biological functions in the brain. A discussion of additional pathway analysis can be found in the **Supplemental Information**.

## DISCUSSION

Intracortical microelectrodes are used for neuroscience research and clinical brain-machine interface systems, but the recording performance decreases over prolonged implantation periods^3^. A major factor in the degradation of implant performance is the neuroinflammatory response^4^. Degradation of BBB integrity is an appreciable consequence of microelectrode-mediated neuroinflammation and can allow previously restricted blood-borne components to enter the brain parenchyma and amplify the neuroinflammatory response^4,6,15–17^.

During disease and injury, constituents of the gut microbiome can directly infiltrate the brain, causing a local inflammatory response^20^. Gut-resident microbiota activate and modulate neuroinflammatory processes implicated in schizophrenia, depression, anxiety, Alzheimer’s, Parkinson’s, and stroke^20,22,23^. Despite this link, there have been no reports examining the infiltration of the gut microbiome following microelectrode implantation and associated device performance. Therefore, the principal hypothesis of the current study was that damage to the BBB caused by microelectrode implantation would amplify dysregulation of the microbiome-gut-brain axis, facilitating the invasion of gut-derived microbes into the brain, escalating the chronic neuroinflammatory response, and contributing to the decreased performance of intracortical microelectrode arrays.

Bacteria can enter the brain at various stages of device implantation during the surgical procedure, ranging from contamination of the initially sterile device to transport by blood to the implantation site^45,46^. However, we have previously shown that after two weeks, residual endotoxin contamination was unable to impact the neuroinflammatory response to intracortical microelectrodes^11^. All implants in this study, control and antibiotic-treated, followed our established protocols to remove bacterial invasion from the microelectrode itself. Therefore, it is unlikely that the responses observed here are due to implant contamination with viable bacteria, or external factors entering the wound margins. Further, several studies in rodents and humans have shown that traumatic brain injuries are accompanied by increased intestinal permeability and intestinal barrier dysfunction^19,47,48^. Therefore, we were particularly interested in understanding the role that microbes that reside in the intestines may have on microelectrode performance if they invade the brain tissue following microelectrode implantation.

In this investigation, we have demonstrated that microbes associated with the gut microbiome invade the brain tissue and reside in sites proximal to the microelectrode implantation site following microelectrode implantation (Fig. 1). To explore whether changes in microbiome composition could affect the profile in the brain, we treated mice implanted with intracortical microelectrodes continuously with an antibiotic cocktail. The antibiotic treatment altered the composition and the abundance of invading microbes in the brain following microelectrode implantation (Fig. 1). The differential composition and abundance of invasive microbes were associated with significant temporal changes in the recording performance of intracortical microelectrodes (Fig. 2), which correlated to dynamic changes in the neuroinflammatory state of the microelectrode-tissue interface (Fig. 3–4). Antibiotic treatment after microelectrode implantation resulted in significantly improved recording performance (Fig. 2), up to 5 weeks post-implantation. While the 12-week post-implantation brain microbe composition more similarly represents the pre-implantation brain microbe environment, it remains significantly different in content and composition. It is important to recognize that even subtle changes in the microbe composition in the brain have been linked to changes in brain health^20,22,23^.

The most abundant microbes identified in brain tissue following microelectrode implantation were Firmicutes and Bacteroidetes, which are the most dominant bacterial phyla in the gut of at least 60 mammalian species^33^. Additionally, microbes not identified in fecal matter or unimplanted brain tissue were found in microelectrode-implanted brain tissue. The brain is a distinct environment from the colon. Therefore, it is not without reason that many invading species are unable to thrive in the brain and are readily removed – dead or alive. Together, our results suggest the microbes are invading the brain tissue following intracortical microelectrode implantation from potentially multiple sources, with a high likelihood of gut-derived sources. To theorize the origin of the invading bacteria, we analyzed whether the invading bacteria of the brain were also detected in the healthy, unimplanted brain (native bacteria), the gut (gut-derived bacteria), or neither (Fig. 2B). While around ~ 28% of invading bacteria can also be found in the gut, ~ 72% originate from a location beside the gut or unimplanted brain, presenting a need to investigate other potential sources of bacteria in the body. However, it is important to note that bacteria of the brain have been poorly characterized, leading to the possibility that there are bacteria in the brain after implantation that are unable to be matched to current databases. The results of the present study demonstrate that the brain bacteria environment is significantly impacted after intracortical microelectrode implantation and that it is possible to influence the type of bacteria present at the microelectrode-tissue interface. The modulation of the bacteria environment is associated with changes in the intracortical microelectrode recording performance and the neuroinflammatory response near the implant site, which has been identified as a major factor influencing microelectrode performance^3–5^.

The acute improvements in microelectrode recording performance reported here, in combination with the alterations to brain microbe composition at 4 weeks, indicate a potential avenue for new therapeutics to improve brain implant function and mitigate neuroinflammation. While not designed to be a solution, the extreme antibiotic treatment utilized here represents a proof of concept for designing a more tailored approach to target specific gut-derived bacteria strains that may be exacerbating the inflammatory response of the brain. For example, at 4-weeks post-implantation, differences in microbe composition and abundance between antibiotic-treated and untreated control groups were largely in the phylum Firmicutes. Specific strains of Firmicutes have been linked to neurodegenerative diseases such as multiple sclerosis, autism, depression, and schizophrenia^49^. The reduction of Firmicutes composition in brain tissue adjacent to the intracortical microelectrode in the antibiotic-treated group may be contributing to the improvements in recording performance through a reduced neuroinflammatory response or even through secondary mechanisms. Recent studies have identified diverse microbiota-derived bioactive molecules that are implicated in inflammatory processes ranging from the gut to the brain^26^. In a pilot study, we examined the fecal matter of a human subject implanted with a brain-machine interface^50^ and found the composition of microbes at the phylum level to be > 90% consistent after human and mouse intracortical microelectrode implantation (**Supplemental Fig. 9**). Therefore, therapeutic approaches designed to provide an optimal balance of invading microbes such as Firmicutes may be beneficial for improving microelectrode recording performance and can be readily tested in mouse models due to the consistency between human and mouse gut microbiome.

Antibiotics are commonly used as part of post-surgery treatments and have been investigated acutely in mice, showing a beneficial reduction of glial encapsulation post-implantation of MEAs^51–53^. However, it is unlikely that regular antibiotic treatment throughout the duration of microelectrode implantation would represent a practical clinical solution to improved microelectrode performance. Long-term dosing of antibiotics is well known to be detrimental to overall health^54^. Chronic administration of antibiotics can lead to the selection of antibiotic resistant bacteria, as well as shift stable, healthy populations of bacteria in the local microbiome into unstable and/or unhealthy ones^55,56^. One strategy that is more commonly employed after prescribing antibiotics is pairing with probiotics^57^. Perhaps, a shorter duration of antibiotic treatment followed by a specific probiotic cocktail to promote the invasion of more benign or even neuroprotective bacteria can be possible with time. Alternatively, the application of antimicrobial coatings to the microelectrode substrate^58,59^ to prevent the population of brain tissue adjacent to the microelectrodes with invasive microbes represents a promising materials-based approach to overcome the newly identified problem. Further investigation into the development of vaccines to regulate T-cell programming^60,61^ towards specific strains of gut-derived microbes, such as Firmicutes, could provide a means to ‘prime’ the adaptive immune system prior to microelectrode implantation.

Interestingly, there were one control and two antibiotic animals at 4 weeks post-implantation that had similar bacterial compositions to that of the 12-week post-implantation brain. Such results may indicate that an animal’s response to treatment and bacterial infiltration may vary, possibly due to their individual immune response or due to the variability reported by many labs in the damage to the BBB following intracortical microelectrode implantation^6,38,62–64^ – either merits further investigation.

It is important to note the limitations of 16S bacteria analysis and its associated assumptions. First, while the work outlined has demonstrated the presence of 16S bacteria at the site of implantation and in the brain, 16S measurement does not confirm the presence of live bacteria and may indicate either dead or fragments of bacterial DNA. The confirmation of live bacteria and the presence of a microbiome in the brain after implantation necessitates further work, including comprehensive live bacteria culture of implanted brain tissue. Second, 16S sequencing results provide a view of bacterial composition and relative abundance, which is not indicative of total bacterial quantity. Lastly, translocation of intestinal bacteria, particularly anaerobic bacteria, occurs infrequently^65^, raising the question of whether the identified 16S bacteria here are metabolically active and growing. However, with the rise of 16S sequencing, previous teachings of anaerobic bacteria translocation may change. To better investigate the effect of bacteria infiltration and antibiotic treatment, we explored proteomic and transcriptomic analysis around the implant site.

Cell-specific spatial proteomic and spatial whole transcriptome analysis revealed that antibiotic treatment impacted dozens of proteins and hundreds of genes at both 4- and 12-weeks post-implantation. We postulate that the large number of downregulated proteins in the antibiotic-treated group at 4-weeks post-implantation may influence a more favorable environment for improved neural recording quality. Many proteins involved with macrophage and microglial response were downregulated (MerTK, CD40, CD68, TFEB), which may account for a reduced neuroinflammatory response and improved recording performance^6,66^. Additionally, transcriptomics revealed overexpression of genes associated with ribosomal subunit structures at 4-weeks post-implantation in the antibiotic-treated group compared to the control, but a majority downregulated at 12-weeks post-implantation in antibiotic compared to the control. Ribosomal dysfunction is commonly associated with neurodegenerative diseases such as Parkinson’s and Alzheimer’s, which may be tied to the switch from significantly improved recordings to worse recordings at those respective time points^67,68^. Proteins associated with autophagy and neural health were downregulated at 4-weeks post-implantation as well (ATG12, SYP, MAP2, NfL, NeuN). Such protein downregulation may be a precursor to the significant drop in implant function observed at week 7 and onward, as loss of autophagy and neural health are often associated with neurodegenerative diseases such as Parkinson’s disease^69^. This is reflected both in the decline of implant function and uptick in differentially expressed genes of the neurodegenerative pathway at 12-weeks post-implantation (49 genes) vs 4-weeks post-implantation (17 genes), including higher dysfunction in mitochondria, UPS disruption for clearing misfolded proteins, and tau protein accumulation, all of which are common indicators of neurodegeneration and disease states^70–72^. Continuing to develop an in-depth understanding of changes in gene and protein expression following changes in invasive microbe composition could identify novel pathways for molecular or gene therapy^73^ approaches to modulating the innate immune response following intracortical microelectrode implantation.

## CONCLUSION

The results of the current study indicate that normally gut-resident microbes and microbes of a currently unknown origin can invade the brain after intracortical microelectrode implantation. Further, it is possible to modulate the neuroinflammatory response following implantation and microelectrode performance by altering the composition and abundance of invasive microbes. The importance of microbes invading the brain extends far beyond device performance and tissue reaction alone and raises concerns about unintended consequences or ripple effects. Some of the microbial strains identified in brain tissue in this initial study have been previously associated with neurodegenerative symptoms and diseases. This raises long-term concerns and requires the development of a comprehensive approach for the optimal integration of neuro-modulatory devices within the brain tissue. While the focus of the current study was solely intracortical microelectrodes arrays, devices with a larger footprint could presumably produce an even more pronounced effect if, in fact, the nature and extent of BBB damage determines the microbial invasion of the brain. Future studies should further investigate both the mechanism of invasion and approaches to mitigate the invasion and colonization of the brain by gut-derived microbes.

## MATERIALS AND METHODS

All procedures and animal care protocols were performed in compliance with Case Western Reserve University’s Institutional Animal Care and Use Committee (IACUC) approved protocol.

### Intracortical Microelectrode Arra>y Preparation

A 16-channel single-shank intracortical microelectrode array (A1×16-3mm-50-177-Z16, iridium electrode sites, NeuroNexus Technologies, Ann Arbor, MI, USA) was used to record neural action potentials of the motor cortex (M1). Alternatively, a non-functional silicone implant of the same dimension was used to assess neuroinflammation and microbial composition. In a Faraday cage setup, each MEA to be implanted underwent EIS testing with a Gamry Interface 1010E Potentiostat (Gamry Instruments, Warminster, PA, USA) consisting of each electrode site as the working electrode, a platinum wire as a counter electrode, and an Ag|AgCl electrode stored in KCl reference electrode for measurements. EIS was performed in 1x PBS (pH = 7.4) over a range of 1 to 10^6^ Hz (12 points per decade) with an AC voltage of 50 mV. The impedance magnitude at 1 kHz was used to confirm functionality with expected values between 150 – 550 kHz. Following EIS verification, MEAs were cleaned using 70% ethanol and DI water to remove any residual 1x PBS and optically imaged using a Keyence Optical Microscope (Keyence Corporation, Osaka, Japan) at a magnification of 150x for visual inspection. Non-functional dummy implants were cleaned using the same protocol as functional implants. After cleaning, both implant types were sterilized using cold gas ethylene oxide.

### Intracortical Microelectrode Implantation

**Figure 6:**
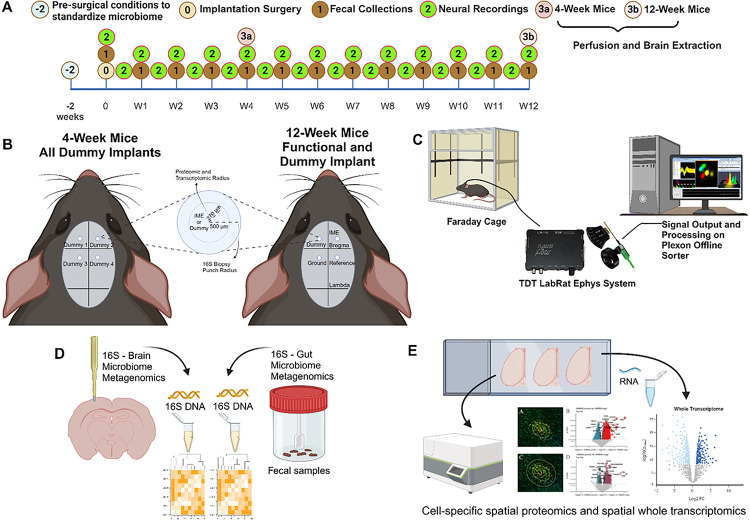
Experimental design outlining the timeline for each cohort. (A) The unimplanted mice were sacrificed two weeks after housing separation for analysis. The 4- and 12-week post-implantation animals undergo implantation, fecal collection, neural recordings, and perfusion at their endpoint. (B) The 4-week cohort receive four non-functional dummy implants and the 12-week cohort receive one non-functional dummy implant and one functional implant with respective ground and reference wires. (C) 12-week functional implanted mice were recorded using a commutator hooked up to the TDT LabRat Ephys system. (D) 16S analysis was done on a biopsy of brain tissue around the implant site and on fresh fecal matter collected from each animal. (E) Cell-specific spatial proteomics and spatial transcriptomics were performed on various brain samples sectioned onto microscope slides.

C57BL/6 mice were obtained from Jackson Labs aged 8–10 weeks and separated to single housing before surgery. Each cohort of animals followed the experimental timeline outlined in Fig. 6A with end points of 4-weeks and 12-weeks post-implantation. All surgical procedures followed established protocols in our combined labs^110,111^. Briefly, mice were anesthetized in an isoflurane chamber (3.5% at 0.8 L/min O_2_). Anesthetic plane was monitored via paw pinch and respiratory rate. Following anesthesia, the incision site was shaved, nails trimmed, and eye lube applied to prevent eyes from drying out. The mouse was then mounted to the stereotaxic frame (David Kopf Instruments, Tujunga, CA, USA) via bite bar and ear bars. Anesthesia was maintained at 0.5% - 2.0% at 0.8 L/min O_2_ via nose cone inhalation. Topical analgesic Lidocaine was applied to the surgical site^66^. Subcutaneous analgesics buprenorphine and meloxicam were administered before surgery. No systemic antibiotic was administered for any group for surgeries. Once mounted, the surgical site was cleaned and sterilized using betadine and 70% isopropyl alcohol in alternating scrubs. A one-inch incision was made along the midline of the scalp and skin retracted using alligator clips to expose the skull. A swab of hydrogen peroxide was applied to the skull to dry out and make cranial sutures more visible. A thin coat of Vetbond tissue adhesive (Catalog #70200742529, 3M, Saint Paul, MN, USA) was applied to the skull to prepare for dental cement adhesion. Using a 1.35 mm drill bit attached to an electric drill, two craniotomies were drilled into the skull to implant, one for the non-functional implant and one for the functional intracortical microelectrodes; two additional craniotomies were made as well for insertion of the ground and reference wires. Using a dura pick, the dura was carefully removed before implantation to expose the implantation site. Mice were divided into two groups, one surviving 12-weeks and a second one 4-weeks post-implantation. The group which survived for 12-weeks post-implantation were implanted with the functional implant inserted 1 mm deep into the primary motor cortex (2 mm anterior to bregma, 2 mm dextral to midline) with reference (2 mm posterior to bregma, 2 mm dextral to midline) and ground wires (2 mm posterior to bregma, 2 mm sinistral to midline) inserted into the brain (Fig. 6B). The non-functional dummy implant was inserted at 2 mm anterior to bregma and 2 mm sinistral to midline. The mice which survived for 4-weeks post-implantation received four non-functional dummy implants inserted at each of the above four coordinate sites (Fig. 6B). Once a wire or implant were inserted, they were secured in place using Kwik-Sil silicone elastomer (World Precision Instruments, Sarasota, FL, USA) to close off the opening of the brain. Following which, Teets dental cement (A-M Systems, Sequim, WA, USA) was applied to anchor the wires and implants to the skull and prevent movement over the course of the study. Following surgery, 5–0 monofilament polypropylene sutures were used to close the surgical site and promote healing of the skin and tissue. A daily dose of analgesic meloxicam and twice daily buprenorphine were administered for 72 hours post-operation to manage pain.

### Treatment and Preparation

To manipulate the composition of the gut microbiome we used a high-dose antibiotic mixture administered to the mice. A mixture of Ampicillin (Millipore Sigma, A5354), Clindamycin (Millipore Sigma, PHR1159), and Streptomycin (Millipore Sigma, S9137) were provided via sterile drinking water at a concentration of 0.33mg/mL for each antibiotic. Such antibiotics were chosen based off previous literature to provide broad spectrum capacity and effect on the gut microbiome^29^. Animals drank *ad libitum* from the water and was replaced every 3 days. Control mice received normal food and water diets. All animals were singly housed in a reversed 12-hour light cycle.

### Neurophysiological Recording and Analysis

Electrophysiological recordings were taken from the functional intracortical microelectrode twice weekly beginning on day 0 of the implant and continuing throughout the duration of the 12-Week implants to assess device function. To record, animals were briefly anesthetized using isoflurane at 3.5% and 0.8 L/min O_2_. While anesthetized, animals were placed into an acrylic box surrounded by a Faraday cage and connected to the recording equipment (Fig. 6C). The functional intracortical microelectrode was connected to a 16-channel ZIF-Clip Headstage (Tucker-Davis Technologies Inc., Alachua, FL, USA) which was part of a 32-channel motorized commutator system (Catalog #ACO32, Tucker-Davis Technologies Inc) for free movement without damaging the wires. The commutator was then connected directly to a Lab Rat Ephys system (Tucker-Davis Technologies) and into a laptop for processing. Using the Synapse recording software (Tucker-Davis Technologies), recordings were taken at a sampling rate of 24414 Hz with a bandpass filter between 300 – 3000 Hz. Recording files were analyzed using Plexon Offline Sorter (Plexon Inc, Dallas, TX, USA) by first converting recordings to a usable. DDT format and importing into Plexon Offline Sorter for single unit analysis. Once imported, common median referencing was performed to reduce noise across channels. If any bad channels were known on the device, as observed during recordings for abnormal noise or activity levels, they were excluded to prevent interfering with the other channels. Once referenced, spikes were detected using settings of −4.00 standard deviation (σ) from the mean with waveform settings of 1720 μsec for waveform length, a pre-threshold period of 410 μsec, and a dead time of 1352 μsec. To remove any possible artifacts that were not filtered out, amplitudes of +/− 500 μV were removed along with any identical spikes that were detected across 90% of the channels. If there were any particularly noisy portions of a recording (e.g. a wire getting caught or the animal interfering with the connection), the noisy intervals were removed using the interval selection tool. If high noise happened excessively during recording, all connections were checked between the device, headstage, commutator, and computer, and the recording was immediately redone. After filtering and detecting spikes, single unit sorting was performed using the K-Means scan algorithm in Plexon Offline Sorter to find between 1 and 4 units on each channel. From here, manual validation was performed on every channel to ensure that all units detected were correctly identified as single units. In many cases, units were deleted as they did not have typical characteristics for single units^112^. From this, the total number of active channels (channels picking up a single unit recording) for each recording was recorded to determine the % of active channels for each animal as a main outcome for recording performance. After manually checking for single units, files were exported and analyzed in MATLAB R2021a (Mathworks, Natick, MA, USA) to calculate peak-to-peak voltage (V_pp_), noise levels, signal-to-noise ratio (SNR), spiking rate, and the number of single units detected per channel. V_pp_ was calculated as the sum of the peak and trough signal of each waveform, noise was calculated at the root-mean-square of the channel after removing spikes, SNR was calculated by dividing V_pp_ by the noise for each unit, and spiking rate was defined as the inverse of the median interspike interval per unit (from Plexon Offline Sorter). To summarize the data, the recording metrics for each individual intracortical microelectrode were averaged in their respective groups and time points. Time points were grouped into 3 phases corresponding to an acute phase (weeks 0 – 5), a sub chronic phase (weeks 6 – 11), and a chronic phase defined as any time points after week 11. Sample size for the week-by-week “Proportion of Active Electrodes” was determined by summing the total number of electrodes multiplied by the number of animals in each group on a week-by-week basis. The sample size for acute, sub-chronic, and chronic “Proportion of Active Electrodes” was determined by summing the total number of electrodes multiplied by the number of weeks in each phase and the number of animals in each group. The sample size for the additional recording metrics was calculated by averaging the respective recording metric on a per-channel basis, and then summing up all unique channels across all animals within the same group and time point (e.g. if channel 1 of antibiotic animal 1 records an SNR on weeks 1, 2, and 3, then those values were averaged into a singular SNR value for channel 1 during the acute phase of antibiotic animal 1 to be used in further analysis).

Using Excel (Microsoft Corporation, Redmond, WA, US), a one-tailed proportions z-test was used for calculating statistical differences in the proportion of active electrodes within and across groups for the acute, sub-chronic, and chronic phases. Additional recording metrics were compared using R Studio 2022.7.1+554 (RStudio, PBC, Boston, MA, USA) and GraphPad Prism (Dotmatics, Boston, MA, USA) within and across acute, sub-chronic, and chronic neuroinflammatory phases using a Kruskal-Wallis test followed by a Benjamini–Krieger–Yekutieli test to adjust for multiple comparisons for non-normal distributions to increase statistical power and reduce type I errors. Statistical comparisons for antibiotic vs. control were only conducted within the same time point (acute antibiotic vs. acute control, sub-chronic antibiotic vs. sub-chronic control, chronic antibiotic vs. chronic control). No comparisons were made across time points and groups (acute antibiotic vs. chronic control, acute antibiotic vs. sub-chronic control, etc.) due to a lack of relevance concerning treatment effect. In all cases, statistical significance was defined at p < 0.05. For recording data box plots, whiskers represent minimum and maximum values, the box represents the first and third quartiles of the data, and the horizontal line indicates the median. All recorded numerical data were represented in the text as the mean ±SD.

### Fecal Matter and Brain Sample Isolation in Mice

Weekly mouse fecal samples were taken from every singly housed mouse to provide samples to measure 16S bacteria of the gut throughout the duration of the study (Fig. 6A). On the day before fecal matter collection, each animal’s housing was changed to fresh, sterile bedding. The next day a microcentrifuge tube of fecal matter was collected using sterile, disposable forceps before being stored in a −80°C freezer until processing. At the end point of the study, animals were perfused to extract brain tissue (Fig. 6A). Animals were injected with an IP anesthetic injection of Ketamine (100 mg/kg) and Xylazine (10 mg/kg). Sufficient anesthetic depth was determined by paw pinch before proceeding with perfusion. Once anesthetized, an incision was made along the abdomen just below the xyphoid process. A horizontal cut was made down the sides of the abdomen proceeded by two vertical cuts through the rib cage on both sides. The rib cage was held up using a pair of hemostats and diaphragm cut through to expose the heart and lungs. Once exposed, a butterfly needle was inserted into the left chamber of the heart and perfusate was pumped through the body. As soon as perfusate was turned on, a small cut was made on the right ventricle to allow for liquid to flow from the heart and prevent collapse of the heart. Approximately 15 mL of each solution was needed to perfuse the animal as indicated by a flushing of the liver. Following perfusion, the brain was extracted, and a biopsy punch was taken around an implant site for analysis. One implant site was biopsy punched for 16S bacterial DNA analysis (Fig. 6B). The rest of the brain and remaining implant sites were left for proteomic and transcriptomic analysis by freezing in a mold containing Optimal Cutting Temperature (OCT, Sakura Finetek USA Inc, Torrance, CA, USA) and placed into a −80°C freezer until processing (Fig. 6D).

### Human Fecal Matter Collection

Human fecal matter was collected under an approved IRB protocol at University Hospitals Cleveland Medical Center in collaboration with the Reconnecting the Hand and Arm to the Brain (ReHAB) clinical trial (clincaltrials.gov #NCT03898804). At the time of sample collection, the study participant (coded RP1) was a 29-year-old male who had suffered spinal cord injury (C3/C4, AIS B), resulting in tetraplegia (motor-complete, sensory-incomplete), 8 years prior. His participation in the ReHAB pilot clinical trial has been previously reported^50^. Briefly, RP1 received six 64-channel (8×8) Utah intracortical microelectrode arrays (Blackrock Microsystems, Salt Lake City, UT) implanted into various sensorimotor cortices for the purpose of restoring cortically-controlled movements of his paralyzed arm and hand, reanimated by functional electrical stimulation through composite flat interface nerve cuff electrodes. RP1 received the cortical implants 2 years and 6 months prior to fecal sample collection. Fecal matter was collected using standard procedures and transported in sterile and sealed containers, packaged in dry ice, for subsequent analysis. Samples were stored in a −80°C freezer until processing until sequencing.

### 16S Bacterial DNA Sequencing

To analyze 16S bacterial DNA, all fecal matter and brain samples were sent to the genomic core on Case Western Reserve University’s campus for DNA isolation and 16S sequencing. The V3-V4 region of the 16S rRNA small subunit (464 base pairs) was sequenced using an Illumina MiSeq (Illumina Inc., San Diego, CA, USA) with a paired-end 250-cycle run. Raw paired-end sequences were processed and assigned to an operational taxonomic unit (OTU) using QIIME2 v2023.5^113^. Sequencing primers were trimmed from the reads and untrimmed sequences discarded using the Cutadapt plugin. Forward and reverse trimmed sequences were joined (minimum overlap = 4 bases) and the merged sequences denoised with the DADA2 plugin^114^. Representative sequences were assigned to operational taxonomic units (OTUs) with a Naïve Bayes classifier trained on the V3-V4 region of 16s rRNA extracted from the SILVA v138.1 SSU (small subunit, 16s/18s rRNA) Ref NR 99 reference sequences using the feature-classifier plugin^115^.

Sequences were queried against the NCBI mouse (GRCm39 assembly, RefSeq accession GCF_000001635.27) and human genomes (GRCh38.p14 assembly, RefSeq accession GCF_000001405.39) as well as the prokaryote 16s rRNA sequences downloaded from the BLAST database (v5) using the rBLAST package v0.99.2^116^. Sequences with hits in the eukaryotic genomes (mouse, n = 834; human, n = 2) as well as sequences with no 16s hit and no OTU assignment from QIIME2 (n = 632) were discarded^116^. No sequences had hits in both the eukaryote and prokaryote databases. Read counts for the remaining sequences (n = 4862) were summed by OTU, filtered for OTUs with more than 5 reads in at least 2 samples (n = 369).

Microbiome data was managed using the microbiome package v1.20.0. The Shannon Diversity Index and total observed features were calculated for each sample rarefied to the lowest sampling depth by sample source (brain or fecal). Differences in these measures and in invading microbe abundance by implantation status and treatment group were assessed via two-way ANOVA using an aligned rank transform for nonparametric factorial analyses^117,118^. An unrooted phylogenetic tree was produced by aligning representative sequences with the DECIPHER package v2.26.0 using the default parameters and de novo assembly with the phangorn package v2.11.1^119–121^. The best nucleotide substitution model, the transition model TIM1+G(4)+I, was selected using the lowest Bayesian Information Criterion among available models and an unrooted tree inferred and optimized via maximum likelihood. Samples were ordinated via non-metric multidimensional scaling on unweighted Unifrac distances calculated using the phylogenetic tree. Differences in the distances by implantation status and treatment group were assessed by PERMANOVA with the vegan package v2.6.4^122,123^. Linear discriminant analysis Effect Size (LEfSe) was used to determine OTUs enriched in brain samples from each implantation status using the microbiome Marker package v1.4.0, with taxa having a linear discriminant analysis score greater than 4.5 being considered enriched^30,124^. The differential abundance of OTUs agglomerated at the phylum through genus levels was assessed with ANCOMBC2 from the ANCOMBC package v2.0.3^125,126^. The Benjamini-Hochberg method was used to adjust p-values of pairwise comparisons for multiple testing. All the analyses were performed in R 4.2.3 in Windows 10 ×64^127^.

### Spatial Proteomic Analysis of the Implant Site

**Figure 7:**
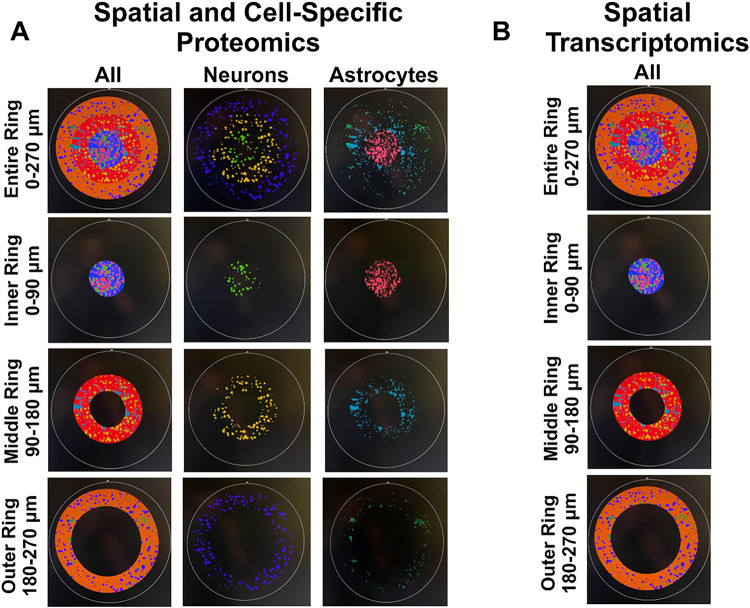
Spatial and Cell-Specific Analysis of the Implant Site Using Proteomics and Transcriptomics. (A) Proteomics analysis was performed on the entire implant ring (0 – 270 μm from the implant site), inner ring (0 – 90 μm from the implant site), middle ring (90 – 180 μm from the implant site), and outer ring (180 – 270 μm from the implant site) on a cell-specific basis for neurons, astrocytes, and all cells. (B) Transcriptomic analysis was not done using cell-specificity. Only spatial separation to analyze the implant regions was performed.

Frozen, non-fixed brains were first sectioned at 5 μm thickness using a cryostat and mounted onto microscope slides (SuperFrost Plus, FisherBrand, Hampton, NH). One section from the middle depth of the implant (~500 μm deep into the cortex) for each brain was taken and sectioned onto each slide. Doing so yielded slides containing one brain slice from each animal in the study. Once slides were prepared containing each brain slice, spatial proteomic analysis was done using the NanoString GeoMx and nCounter suite of equipment and reagents and following their established protocols (NanoString Technologies, Seattle, WA, USA) (Fig. 6E). For proteomic analysis, slides were first submerged in 10% neutral buffered formalin (NBF, Thermo Fisher Scientific, Waltham, MA, USA) for 12–16 hours followed by 3x washes in 1x Tris-Buffered Saline with Triton (1x TBS-T, NanoString). Briefly, slides then undergo antigen retrieval with 1x Citrate buffer using the TintoRetriever Pressure Cooker (Bio SB, Item Number: BSB 7008) on high temperature and pressure settings for 15 minutes followed by blocking tissue for non-specific reaction to antibodies. Morphological antibodies for neurons (1:100 anti-NeuN, Alexa Fluor^®^ 647 EPR12763, Item Number: ab190565) and astrocytes (1:40 anti-GFAP, Alexa Fluor^®^ 532 GA-5, Item Number: NBP2-33184AF532) were then incubated in a humidity chamber overnight in a 4°C refrigerator along with antibodies specific to NanoString mouse neural proteomics panel at 1:25 concentration (**Table 1**). The mouse neural proteomics panel consists of the Neural Cell Profiling Core (25 proteins, Item Number: 121300120) paired with the Glial Cell Subtyping Module (10 proteins, Item Number: 121300125) and the Autophagy Module (10 proteins, Item Number: 121300124). Following overnight primary antibody incubation, tissue was washed three times in 1x TBST for ten minutes each then postfixed with formalin for 30 minutes. Residual formalin was washed off twice in 1x TBST for five minutes each before being stained with a nuclear stain (1:10 Syto13, NanoString Technologies, #121300303) before imaging. Using the NanoString GeoMx, tissue was imaged, and the implant site was identified. Regions of interest were then selected for protein extraction. Here, we extracted proteins from areas stained by either NeuN, GFAP or from the entire AOI in three regions: 0–90 μm from the implant site (inner region), 90–180 μm from the implant (middle region), or 180–270 μm from the implant (outer region) (Figure 7A).

Once collected into the 96-well plate, the plates were dried overnight in the GeoMx at room temperature before being rehydrated in DNAse/RNAse-free water. After, GeoMx Hybridization Codes (NanoString Technologies, Item Number: 121300401) were added to each row A-H to distinguish between each row and allow for pooling of samples. Each column was then pooled to a final collection of 12 pooled sample solutions that were then loaded into the nCounter MAX/FLEX system (NanoString) for barcode analysis to obtain protein expression counts.

Raw proteomic counts from the neural proteomic panel were uploaded and analyzed using a custom MATLAB R2021a script, following previously established protocols^39^. First, the negative and positive spike-in proteins were removed from analysis. From here, all protein counts were normalized to the geometric mean of the housekeeping proteins. Housekeeping proteins were used for normalization due to their prevalence in all samples and accounts for the number of cells and proteins across varying runs. Housekeeping proteins were not included in the differential expression comparisons. The log_2_(fold change) (log_2_(FC)) for each protein was calculated for each comparison. After normalization, unpaired t-tests were performed across respective groups for comparison. Unadjusted p-values were corrected using the Benjamini-Hochberg false discovery rate method to account for random significance. Data was visualized with volcano plots using GraphPad Prism Plus. All proteomic volcano plots show the −log_10_(p_adjusted_) plotted against the log_2_(FC). A dotted line indicates the significance threshold, as determined using the adjusted p-values calculated.

### Spatial Transcriptomic Analysis of the Implant Site

Frozen, non-fixed brains were first sectioned at 5 μm thickness using a cryostat and mounted onto microscope slides (SuperFrost Plus, FisherBrand, Hampton, NH). One section from the middle depth of the implant (~500 μm deep into the cortex) for each brain was taken and sectioned onto each slide. Doing so yielded slides containing one brain slice from each animal in the study. Once slides were prepared containing each brain slice, spatial proteomic analysis was done using the NanoString GeoMx and nCounter suite of equipment and reagents and following their established protocols (NanoString Technologies, Seattle, WA, USA) (Fig. 6E). For transcriptomic analysis, 0–90 μm from the implant site (inner region), 90–180 μm from the implant (middle region), or 180–270 μm from the implant (outer region) were analyzed; there were no cell-specific transcriptomics performed (Figure 7B). Slides were again fixed overnight in 10% NBF followed by 3x washes in 1x PBS and sequential washes in 50% ethanol, 70% ethanol, and 100% ethanol. From here, antigen retrieval was performed using 1x Tris-EDTA (NanoString) for 20 minutes. RNA targets were then exposed using Proteinase K (NanoString) at a concentration of 1 μg/mL for 15 minutes before undergoing a postfix to preserve tissue morphology using NBF. An overnight in situ hybridization step then occurs to bind the RNA probe mix to RNA targets on tissue. The probe mix used here contains the whole transcriptome atlas for mouse tissue utilizing NanoString’s barcode identification technology (NanoString Catalog: GeoMx RNA WTA Mm). Following hybridization, tissue was washed with a mixture of 100% formamide (NanoString) and 4x Saline Sodium Citrate buffer (4x SSC, NanoString) to remove any off-target probes. Morphology markers for GFAP and NeuN were then added along with SYTO 13 for visualizing the implant site during imaging. From here, regions of interest of 0–90 μm (inner), 90–180 μm (middle), and 180–270 μm (outer) around the implant site were selected for extracting RNA. Once extracted, RNA for the whole transcriptome was sent to the Case Western Reserve University genomics core for sequencing using the Illumina NextSeq 550. After sequencing, FASTQ files were loaded into NanoString’s NGS pipeline software to convert into DCC before processing using the GeoMx software suite.

First, technical and biological quality control was performed to remove any outlier genes and genes with minimal expression detected. Filtering was also performed to remove any genes that did not show expression in at least 5% of the analyzed segments. Quality control and filtering parsed the data down from 20175 genes to 8272 genes for analysis. For measuring the entire AOI region, the inner, middle, and outer regions were summed together on a per sample basis before proceeding to normalization. Each gene underwent Q3 normalization followed by statistical analysis using a custom MATLAB R2021a script to perform unpaired t-tests between samples. Unadjusted p-values were used for all further comparisons in the iPathways software suite. Volcano plots were created using GraphPad Prism Plus 10 and include the −log_10_(unadjusted p-value) plotted against the log_2_(fold change) for each gene. The dotted line indicates significance.

## Figures and Tables

**Figure 5:**
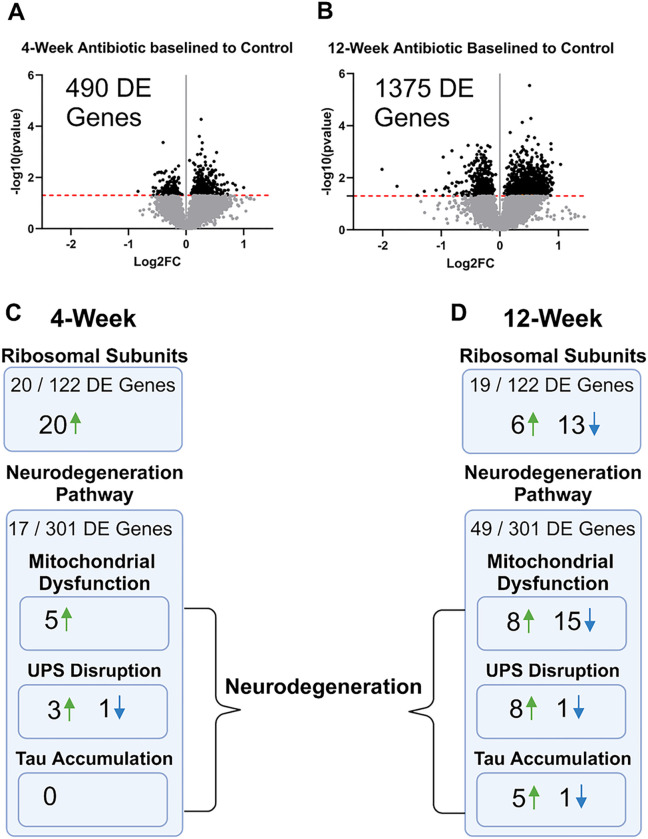
Spatial Transcriptomics Reveals Treatment- and Time-Dependent Effects After Implantation. Transcriptomic data composing the full AOI of the implant site (within 270 μm from the implant site). Volcano plots are shown evaluating gene expression at 4- and 12-weeks post-implantation. Unadjusted p-values are plotted and shown. The black dotted line indicates significance (p_value_ = 0.05). Each point on the volcano plot indicates a singular gene. There were (A) 490 differentially expressed (DE) genes at the 4-week time point between antibiotic and control, which increased to (B) 1375 DE genes at the 12-week time point. Some pathways of note that were impacted by the antibiotic treatment at (C) 4-weeks post-implantation include the ribosomal subunit structure and neurodegeneration pathways, with changes occurring temporally as seen (D) in the 12-week time point.
